# Pathophysiology of Hip Disorders in Patients with Mucopolysaccharidosis IVA

**DOI:** 10.3390/diagnostics10050264

**Published:** 2020-04-29

**Authors:** Zhigang Wang, Yunlan Xu, Enze Jiang, Jianmin Wang, Shunji Tomatsu, Kaiying Shen

**Affiliations:** 1 Department of Pediatric Orthopedics, Shanghai Children’s Medical Center affiliated to Shanghai Jiao Tong University School of Medicine, Shanghai 200127, China; 2 Department of Hematology/Oncology, Shanghai Children’s Medical Center affiliated to Shanghai Jiao Tong University School of Medicine, Shanghai 200127, China; 3 Departments of Orthopedics and BioMedical, Skeletal Dysplasia, Nemours/Alfred I. duPont Hospital for Children, Wilmington, DE 19803, USA

**Keywords:** mucopolysaccharidosis IVA, femoral head osteonecrosis, hip dysplasia, cartilaginous characteristics, magnetic resonance imaging

## Abstract

Patients with mucopolysaccharidoses IVA (MPS IVA) have a progressive accumulation of the specific glycosaminoglycans (GAGs): chondroitin-6-sulfate (C6S) and keratan sulfate (KS), leading to the degeneration of the cartilage matrix and its connective tissue perturbing the regular microarchitecture of cartilage and successively distorting bone ossification and growth. Impaired cartilage quality and poor bone mineralization lead to serious hip disorders in MPS IVA patients. Although hip dysplasia is seen widely in musculoskeletal abnormality of this disorder, the pathophysiology of the hip bone and cartilage morphology in these patients remains unclear. Until now, no systemic study of the hip joints in MPS IVA has been reported by using the combined images of plain film radiographs (PFR) and Magnetic Resonance Imaging (MRI). This study aimed to assess the bony and cartilaginous features of hip joints and to explore the potentially related factors of femoral head osteonecrosis (FHN) and hip subluxation/dislocation in patients with MPS IVA. Hip joints in MPS IVA patients were retrospectively reviewed, based on the findings of PFR and MRI data from 2014 to 2019. Demographic information was also collected and analyzed with imaging measurements. A total of 19 patients (eight boys and 11 girls) were recruited, and 38 hip joints in these patients were examined. Eleven patients (57.9%) had FHN. FHN patients were statistically compared with those without FHN. Correlations between cartilaginous femoral head coverage (CFHC) and acetabular index (AI), cartilaginous AI (CAI), or neck-shaft angle (NSA) were investigated in patients with hip subluxation or dislocation. The greater cartilaginous coverage of the hips than their osseous inherency was observed. Significant correlation was observed between CFHC and AI (r =−0.351, *p* = 0.049) or CAI (r =−0.381, *p* = 0.032). Severe subluxations or dislocations were more likely to be present in those with more dysplastic bony and cartilaginous hips. In conclusion, our study provides the first systemic description of bony and cartilaginous characteristics in the hip morphology of MPS IVA patients. We have demonstrated that plain radiography alone leads to a misunderstanding of hip morphology and that MRI measurements with PFR are an essential tool to evaluate the ‘true’ characterization of hips for MPS IVA patients.

## 1. Introduction

Morquio A syndrome (Mucopolysaccharidosis (MPS) IVA (MPS IVA)) is a lysosomal storage disease caused by the deficiency of N-acetylgalactosamine-6-sulfate sulfatase (GALNS) [1]. This enzyme deficiency leads to the accumulation of two specific glycosaminoglycans (GAGs): chondroitin-6-sulfate (C6S) and keratan sulfate (KS), which results in progressive systemic skeletal dysplasia. Patients with MPS IVA were first described simultaneously in 1929 by Morquio and Brailsford [2,3]. Clinical features typically include disproportionate dwarfism with short neck, hearing loss, fine corneal clouding, marked ligamentous laxity, and systemic skeletal deformity [4,5].

Musculoskeletal disorders almost occur in all types of MPS; however, patients with MPS IVA have unique clinical features compared with other types of MPS, since the skeletal abnormality is more dominant with the laxity of joints [6]. Hip abnormalities, including hip dysplasia, femoral head osteonecrosis (FHN), and coxa valgus, are most common and progressive with age in these patients. The characterization of hip morphology had been described in several types of MPS by plain film radiographs (PFR) [7,8,9,10,11,12,13,14,15]. However, most studies are sporadic cases, and there has been no report of hip assessment by using magnetic resonance imaging (MRI). In 2016, Breyer et al. [16] first reported hip dysplasia pathology in 18 patients with MPS I by using MRI, and have demonstrated that the cartilaginous coverage of the hip was increased compared with that of healthy children and that the use of MRI provided the proper morphology of the hip dysplasia. However, until now, there has been no other systemic MRI study on hip disorders in MPS patients, including MPS IVA. In MPS IVA, Kanazawa et al. (2001) described the sibling case with femoral head dysplasia evaluated by PFR [17]. Management of hip pathologies in these patients is controversial because of progressive FHN. Surgical timing and indication for hip pathologies remain unclear. There were several reports that patients with painful subluxation are candidates for hip reconstruction, and that total hip arthroplasty is an option for young adult patients [18,19,20]. Full understanding of hip morphology for the MPS IVA population can assist physicians in making better treatment decisions to provide a better quality of life to the patients.

In this study, by using the combined images of PFR and MRI, we have aimed to assess the bony and cartilaginous features of hip joints and to explore the potentially related factors of FHN and hip subluxation/dislocation in patients with MPS IVA.

## 2. Materials and Methods

Confirmed MPS IVA patients with established biochemical and genetic identification were retrospectively reviewed between 2014 and 2019 from our databases. All patients were Chinese and were recruited at Shanghai Children’s Medical Center in China. The inclusion criteria consisted of available plain film radiographs and MRI of the hips using standard techniques. Demographic and clinical information included sex, age, age at hematopoietic stem cell transplantation (HSCT), height, and weight. Informed consent was obtained, and institutional review board approval was acquired (SCMCIRB-K2019021-2, approval date: Feb. 2019).

### 2.1. Radiographic Measurements and Analysis

**Figure 1:**
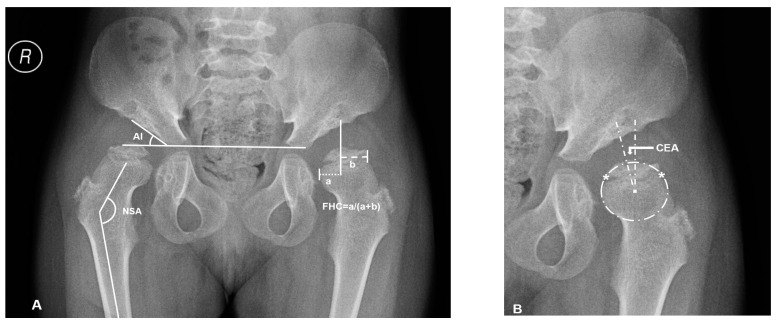
Overview of plain radiographic parameters in a patient with mucopolysaccharidosis (MPS) IVA (No.3: a 6.2-year-old female patient). (**A**) Right hip measurement of the acetabular index (AI) and neck shaft angle (NSA), left hip measurement of femoral head coverage (FHC): ‘a’ is the distance between osseous lateral margin of acetabulum and medial margin of femoral head; ‘b’ is the distance between osseous lateral margin of acetabulum and lateral margin of femoral head. (**B**) The method of Siddesh to define the center of incongruity head. Reference points ‘*’ are marked on the medial and lateral limits of the growth plate. A circle is marked connecting both the reference points, ensuring that the circle just touches the inner margin of the epiphysis but does not extend outside the femoral head. The center of this circle is marked, and then center-edge angle (CEA) is measured.

Supine anteroposterior pelvic plain film radiographs (PFR) with the hips in the neutral rotation was performed in all the patients. The acetabular index (AI), center-edge angle (CEA) of Wiberg, femoral head coverage (FHC, percentage of the femoral head covered by the acetabulum) [21], and neck-shaft angle (NSA) were measured for the evaluations of osseous morphology (Figure 1). FHN was also recorded.

**Figure 2:**
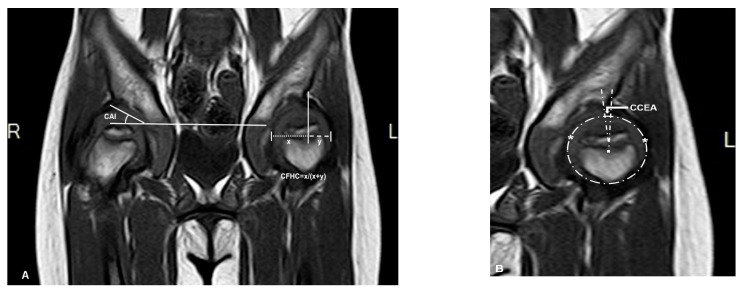
Magnetic resonance imaging (MRI) (coronal T1 sequence) scan of the same patient (patient 3). (**A**) Right hip measurement of the cartilaginous acetabular index (CAI), left hip measurement of cartilaginous femoral head coverage (CFHC): ‘x’ is the distance between cartilaginous lateral margin of acetabulum and medial margin of femoral head; ‘y’ is the distance between cartilaginous lateral margin of acetabulum and lateral margin of femoral head. (**B**) Using the method of Siddesh to measure the cartilaginous center-edge angle (CCEA) of the left hip, ‘*’ is the reference points which are marked on the medial and lateral limits of the growth plate. A circle is marked connecting both the reference points ensuring that the circle just touches the inner margin of the epiphysis but does not extend outside the femoral head [22].

Magnetic resonance imaging (MRI) scans were performed with a maximal slice thickness of 3 mm under sedation. Measurements in MRI imaging included the same angles measured on the PFR (AI, CEA, and FHC). These parameters were measured under the consideration of the cartilaginous roof (CAI, CCEA, and CFHC), which were used to describe the cartilaginous morphology of hips. The measurement center of the femoral head was defined by the Siddesh method [22]. The largest diameter of the head in the coronal view was chosen for measurement (Figure 2).

The differences between osseous and cartilaginous AI, osseous and cartilaginous CEA, and osseous and cartilaginous FHC were calculated and recorded as ∆AI, ∆CEA, and ∆FHC, which represented the ‘true’ cartilaginous characteristics and coverage of the femoral head.

Descriptive statistics were performed for all the included patients. Patients were divided into two groups, according to whether FHN was present or not. The patients’ age, age at HSCT, height, weight, AI, CEA, FHC, CAI, CCEA, CFHC, ∆AI, ∆CEA, and ∆FHC were statistically compared between the two groups.

The patients with subluxation or dislocation of hips, negative value of CCEA, were selected. The extent of subluxation/dislocation of hips was indicated by the values of CFHC. The correlations of CFHC to AI, CAI, and NSA were statistically analyzed.

### 2.2. Statistical Analysis

Data from measured parameters were expressed as mean ± standard deviation (x ± s). We compared the data between the FHN group and the non-FHN group by using the Fisher exact tests for categorical variables and independent t-tests for quantitative variables. Spearman’s 2-tailed correlation analysis was used to test the correlations of CFHC to AI, CAI, and NSA in subluxation/dislocation patients. We analyzed the data using SPSS version 18 (SPSS Inc., Chicago, IL), and the significance level was set at *p* < 0.05.

## 3. Results

### 3.1. Assessment of Bony and Cartilaginous Morphology in MPS IVA Hips

**Figure 3:**
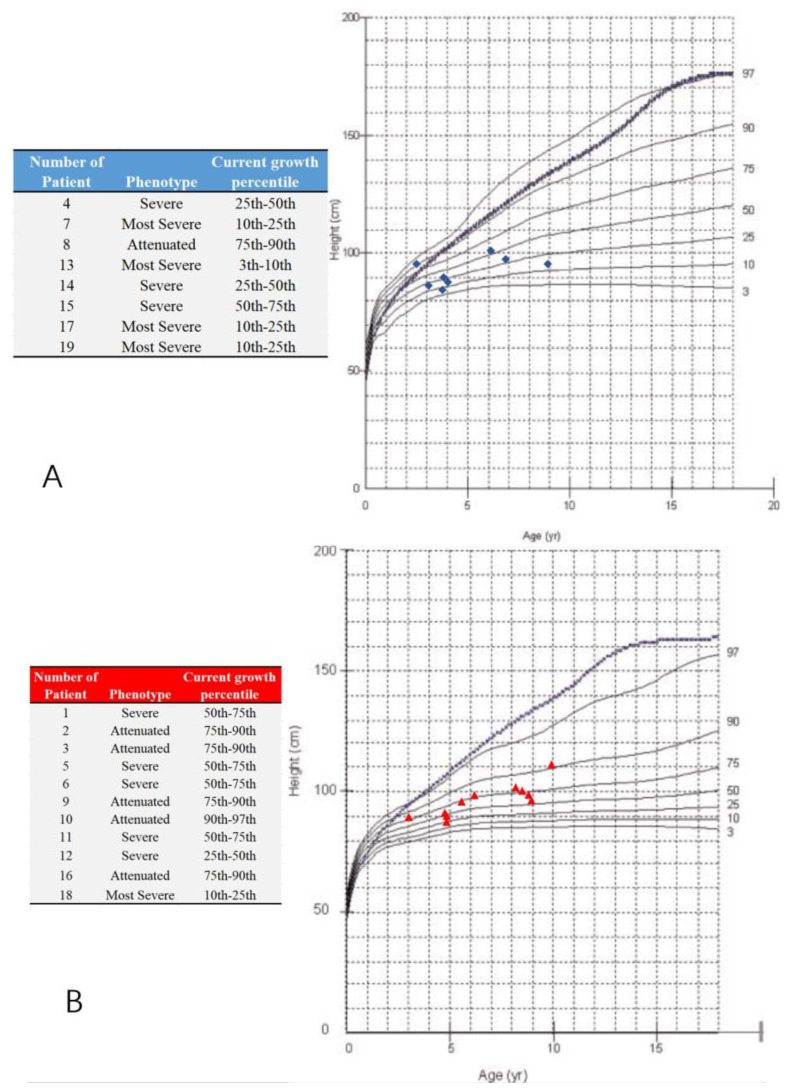
Growth charts of males and females with mucopolysaccharidosis IVA and study participants. (**A**) The blue ‘◆’ shows male participants; (**B**) the red ‘▲’ shows female participants; Note: the dotted and blue lines show the 50th percentile values for normal males and females; revised from Montaño et al. [26]. The phenotype is defined by the present height; less than 25th percentile (the most severe), the 25th–75th percentile (severe), over 75th percentile (attenuated).

**Figure 4:**
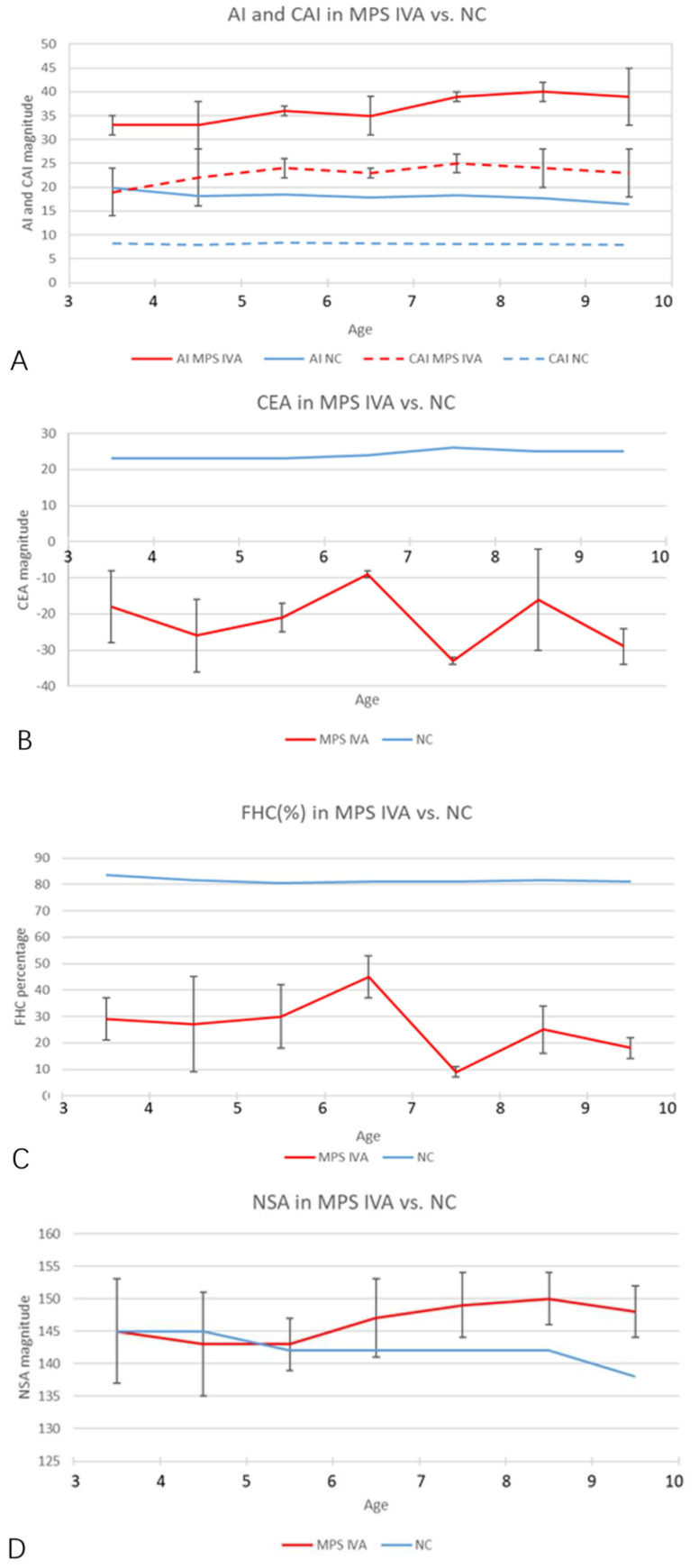
Age-matched comparison of each imaging parameter over 3 years old. (**A**) The AI and CAI magnitudes in MPS IVA hips (red and blue solid lines) increased significantly, compared with normal controls (red and blue dotted lines). Normal values of AI and CAI were cited from Li et al. [23]. (**B**,**C**) The CEA and FHC magnitudes in MPS IVA patients (solid red lines) decreased significantly, compared with normal controls. Normal values (solid blue lines) were cited from Tsukagoshi et al. [24]. (**D**) NSA (solid red line) in MPS IVA patients increased after 5 years old. Normal values (solid blue line) were cited from Birkenmaier et al. [25]. Note: NC, normal control.

A total of 19 MPS IVA patients (eight boys and 11 girls) were included in this retrospective study. The data from 38 hip joints of these patients were collected and investigated (Table 1). All 19 patients were treated with HSCT and had MRI data of both hip joints at 12 months post-HSCT. The mean height was 94.6 ± 15.5 cm (range, 84 to 111 cm), and the mean weight was 6.7 ± 2.5 kg (range, 12 to 21.5 kg). The mean age at the time of imaging, both PFR and MRI, was 6.2 ± 2.2 years old (range, 3 to 9.8 y), and the mean age at HSCT was 5.2 ± 2.2 years old (range, 2 to 8.8 y). The phenotype of each patient was identified by the growth charts of males and females with mucopolysaccharidosis IVA (Figure 3). No patient underwent a previous pelvic or femoral osteotomy before HSCT. CFHC, AI, CAI, CEA, CCEA, and NSA were measured and analyzed. On the PFR, the mean AI was 35.8 ± 4.7 degrees (range, 26 to 46 degrees). The mean CEA was −21.3 ± 11.4 degrees (range, −39 to 7 degrees). The mean FHC was 26.9 ± 14.0% (range, 0% to 56%). The mean NSA was 145.9 ± 6.6 degrees (range, 132 to 156 degrees). In the aspect of MRI measurement, the mean CAI was 22.3 ± 4.7 degrees (range, 10 to 30 degrees). The mean CCEA was −10.8 ± 14.8 degrees (range, −50 to 18 degrees). The mean CFHC was 47.1 ± 10.9% (range, 27% to 65%). Differences between radiologic evaluations and MRI findings were observed in AI, CEA, and FHC. The mean ∆AI, ∆CEA, and ∆FHC were 13.4 ± 5.2 degrees (range, 4 to 25 degrees), 13.1 ± 9.4 degrees (range, 0 to 37 degrees), and 20.6 ± 11.6% (range, 0% to 38%), respectively. Each imaging parameter was compared between the patients studied here and the age-matched normal controls in the literature [23,24,25] (Table 2, Figure 4).

### 3.2. Comparison of FHN and No FHN Patients

FHN in MPS IVA hips is a pathologic process that results from the dysplasia of bone and cartilage by growth disturbance. In this study, eleven patients (57.9%, 11/19) were presented with FHN and eight patients without FHN at the time of imaging (Table 3). No significant difference was found between the FHN and non-FHN groups in terms of sex, CEA, FHC, CAI, CCEA, CFHC, ∆AI, ∆CEA, and ∆FHC. The patients with FHN were significantly older in both age at the imaging and age at HSCT (*p* = 0.002) and were significantly higher and heavier than those without FHN (98.3 ± 5.0 vs. 89.6 ± 5.4 cm, *p* = 0.002 and 16.9 ± 2.2 vs. 13.7 ± 1.7 kg, *p* = 0.003). The AI value in the PFR was significantly larger in the FHN group (37.5 ± 4.0 vs. 33.4 ± 4.5, *p* = 0.007).

### 3.3. Correlated Factors of Subluxation/Dislocation Hips

There were 15 patients with subluxation/dislocation of hips (see ‘*’ in Table 1). Reverse correlations were observed between CFHC and AI (r = −0.351, *p* = 0.049), and CFHC and CAI (r = −0.381, *p* = 0.032). No significant correlation was found between CFHC and NSA (r = −0.148, *p* = 0.418).

### 3.4. Pathophysiology of Hip Disorders in Patients with MPS IVA

**Figure 5:**
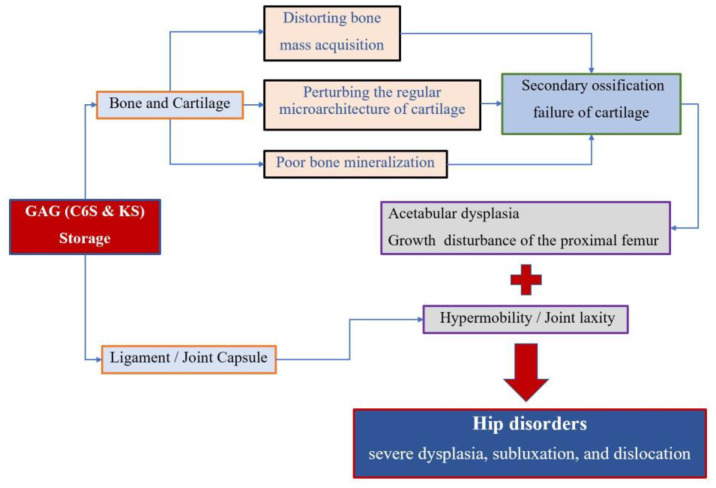
Pathophysiology of hip disorders in MPS IVA.

Accumulation of the glycosaminoglycans (GAGs) (C6S and KS) leads to the degeneration of the cartilage matrix and its connective tissue, perturbing the regular microarchitecture of cartilage and successively distorting bone ossification and growth. MPS IVA patients present impaired cartilage quality because of distortion of geometric shape, collagen disposition in the extracellular matrix (ECM), and remodeling, resulting in reduced bone mineralization [4,27]. Secondary ossification centers of hips are disordered in development, which leads to growth disturbance of the proximal femur and acetabular dysplasia. On the other hand, GAG accumulation in the ligament and capsule of the hips results in hypermobility and joint laxity, which aggravates the instability of hips. All these factors contribute to the molecular pathophysiology of hip disorders for MPS IVA patients (Figure 5).

## 4. Discussion

In this study, we have demonstrated: (1) that the bony and cartilaginous features of the hip joints in MPS IVA are assessed more accurately by using the combined images of PFR and MRI; (2) that the cartilaginous coverage of the hip is higher than the bony inherent in those with severe hip dysplasia; (3) that severe subluxations or dislocations are present in those with more dysplastic bony and cartilaginous hips with reverse correlations between CFHC and AI or between CFHC and CAI; and (4) that the patients with FHN were older, higher, and heavier than those without FHN.

Skeletal and joint abnormalities are the most apparent and prevalent clinical manifestations in MPS IVA patients [28,29]. The GALNS enzyme deficiency leads to a progressive accumulation of the GAGs (C6S and KS), which brings alterations in the connective tissue and cartilage ground substance, distorting bone mass acquisition and perturbing the cartilage microarchitecture [1]. The MPS IVA patients, therefore, present with impaired cartilage quality, namely distorted cartilage geometric shape, abnormal collagen distribution, and cartilage remodeling [4,27]. All these factors mentioned above contribute to the molecular pathophysiology of hip disorders for MPS IVA patients [30,31] (Figure 5). Severe hip dysplasia is common in MPS IVA patients, causing severe pain and successive immobility. However, no accurate assessment of hip dysplasia in MPS IVA has been reported, since only the PFR assessment took place. This is the first report to describe the radiographic characteristics of hip morphology in patients with MPS IVA by using the combined images of PFR and MRI. The principal findings of the present study are the inconsistent manifestations of hip dysplasia between bone and cartilage inherence. Similar consequences were reported in Hurler syndrome (MPS IH) patients by Breyer et al. [16]. Their results of nine MRI and three arthrogram findings established that MPS-IH patients exhibit much greater cartilaginous coverage of the hips. Our results also identified the increased cartilaginous acetabular morphology and head coverage in terms of AI, CEA, and FHC by contrasting the measurements+ of plain radiography and MRI, especially for those severe dysplasia subluxation/dislocation hips. Our study showed an average of about 13 degrees difference between bony and cartilaginous AI, 13 degrees difference between bony and cartilaginous CEA, and a 21% difference between bony and cartilaginous FHC, respectively. It was implied that plain radiography alone leads to a misunderstanding of hip morphology, and MRI measurements are an essential tool to evaluate the ‘true’ characterization of hips for MPS IVA patients.

FHN is a common manifestation of hip disorders in MPS IVA patients [1,18,29]. Eleven (57.9%) patients were identified for presenting FHN in this study. The data also showed the significant difference between FHN and non-FHN patients was the age, age at HSCT, height, weight and AI in plain film radiography. However, we cannot conclude that HCST is beneficial to prevent or delay the FHN without further analysis since the status of the femoral head before HSCT was unavailable. In this retrospective study, the reason that the age at the imaging and the age at HSCT were different in two groups could be due to the age-related pathological progression of skeletal invasion in MPS IVA, rather than the beneficial effect of HSCT. The previous studies reported that progressive collapse and flattening of the ossific nucleus of the proximal femoral epiphyses are observed over time, and ERT had provided no or little benefit for skeletal deformities [1,19,32,33]. In the same way, a larger AI value in X-ray, representing a more dysplastic hip, is more likely to be present in those with FHN with severe hip disorders. Thus, we believe that older patients are more likely to have more dysplastic hips and higher incidence of FHN, which is in accordance with previous reports [5,19]. Generally speaking, an individual with a more severe condition has a more invasive skeletal pathological changes, showing a shorter stature in MPS IVA patients; however, higher and heavier patients were found in the FHN group. We infer that the difference was related to the age bias of patients’ inclusion.

Because of the excessive cartilaginous coverage of the MPS IVA hips, we used CCEA as cartilaginous parameters for the accurate identification for subluxation/dislocation patients. For patients with a ‘-’ value of CCEA, it was indicated that their hip instability would still exist, even if cartilage was considered. For the same reason, the extent of dislocation was represented by CFHC for measuring cartilaginous coverage of head directly. The AI reflected the bony morphology of acetabula, and CAI represented the cartilaginous morphology of acetabula. In all our patients with subluxations/dislocations, we found a correlation between CFHC and AI; and also, between CFHC and CAI; however, we found no correlation between CFHC and NSA. It was implied that the extent of hip instability was related to both bony and cartilaginous acetabula dysplasia, and had no correlation with coxa valgus in MPS IVA patients. In addition to the above bony or cartilaginous factors, including AI, CAI, and NSA at the hip joint, ligament laxity cannot be ignored in MPS IVA patients [2,28,34]. It is difficult to quantify the severity of laxity and explore its correlation with hip instability [35]; however, subluxation/dislocation of the hip joint might be related to the dominant laxity of ligament and joint for MPS IVA. If so, consideration of hip reconstruction with capsulorraphy may be of benefit to the MPS IVA hips.

### Strengths and Limitations

One of the strengths of this study is the fact that we are the first to describe the bony and cartilaginous characteristics of MPS IVA hips in detail. The inconsistency between bone and cartilage was identified in MPS IVA hips. There is also increased cartilaginous coverage of the hip in specific patient populations. The evaluation of cartilage should be considered. Hip MRI is now getting a standard procedure to assess the accurate condition for many bone diseases. In our hospital, all MPS patients with skeletal dysplasia have received hip MRI in combination with the plain film as a clinical indication.

This study also had several limitations. First, it was a single-center study with relatively small sample size and a single image per patient. It is not quite representative of the overall situation of the MPS IVA population. Second, the data of the age-matched normal controls from the literature, which came from a different country, may lead to bias by the difference of racial populations. Third, fewer positive results were obtained. Although our purposes were to explore the potentially related factors of femoral head osteonecrosis and hip subluxation/dislocation in MPS IVA hips, the expected results were not obtained, due to the limitations of retrospective studies. Significant differences were found between FHN and non-FHN patients in the age of HSCT and AI in plain film radiography; however, the conclusion cannot be generalized because of the retrospective study. Similarly, the correlation of hip instability and bony/cartilaginous acetabula dysplasia was shown, but we could not investigate the influence of ligament laxity on MPS IVA hips. The further longitudinal study with multiple points with age is required to clarify the correlation between many factors contributing to hip diseases in MPS IVA.

The appropriate treatment of MPS IVA patients remains controversial since ERT does not provide any impact on bone dysplasia with the high cost. HSCT has been already approved by Japanese (1995) and Chinese (2005) governments much earlier than ERT. Over 25 years, MPS IVA patients have been treated with HSCT until now. Recently, insurance companies in the USA also insured HSCT for MPS IVA patients, and HSCT has been conducted. There are two international guidelines for MPS IVA. One is sponsored by the pharmaceutical industry [36]. Meanwhile, there is another international guideline related to the indication of HSCT in MPS, including MPS IVA, sponsored by non-profit organizations [37]. The latter international group agrees to apply HCT to MPS IVA according to the therapeutic effect; reduction of surgical intervention, better ADL score compared with untreated patients, improvement of joint movement, etc.). In addition, we have not experienced any mortality in our HSCT group of MPS IVA patients. It is critical to have the international guideline not sponsored by the pharmaceutical industry with any conflict of interest. Some governments do not allow any pharmaceutical sponsored guidelines, to avoid any conflict of interest [38]. It is urgently required to have such consensus internationally. We are also investigating the therapeutic effect of these patients treated by HSCT for long-term observation.

## 5. Conclusions

We have demonstrated that MPS IVA patients exhibit significantly higher cartilaginous coverage of the hips than their osseous inherency. Evaluation by plain radiography alone is inadequate for the MPS IVA hips. MRI can be considered as a further modality to assess the ‘true’ characteristics of hips before surgical intervention. Severe subluxation or dislocation is more likely to be present in more dysplastic bony and cartilaginous acetabula for MPS IVA hips. The risk factors of FHN should be further investigated for the MPS IVA patients.

## Figures and Tables

**Table 1:** Characteristics of patients with mucopolysaccharidosis IVA (MPS IVA).

NO.	Sex	Age (y)	Age at HSCT (y)	Height (cm)	Weight (kg)	Phenotype	FHN	AI(°)		CEA(°)		FHC(%)		NSA(°)		CAI(°)		CCEA(°)		CFHC(%)		∆AI		∆CEA		∆FHC	
								L	R	L	R	L	R	L	R	L	R	L	R	L	R	L	R	L	R	L	R
1 *	F	4.9	3.9	91	13.8	S	Y	39	32	−25	−33	11	12	137	135	16	23	−9	−13	40	36	23	9	16	20	29	24
2 *	F	8.4	7.4	100	17.8	A	Y	40	38	−39	−29	14	12	152	154	16	27	−19	−24	27	29	24	11	20	5	13	17
**3**	F	6.2	5.2	98	16.9	A	Y	35	29	−9	−8	40	40	143	153	24	22	11	9	54	64	11	7	20	17	14	24
4 *	M	7.6	6.6	98	18.7	S	Y	39	38	−33	−32	7	10	152	145	26	23	−17	−10	32	48	13	15	16	22	25	38
5 *	F	5.6	4.6	96	15.9	S	Y	35	36	−24	−18	21	38	146	140	22	25	−10	−14	48	44	13	11	14	4	27	6
**6**	F	8.8	7.8	96	15	S	Y	38	42	−20	−18	23	22	154	151	23	28	17	5	65	56	15	14	37	23	42	34
7 *	M	9.7	8.7	96	15.9	S	Y	33	35	−33	−33	19	12	150	148	28	21	−50	−48	28	27	5	14	17	15	9	15
**8**	M	3.2	2.2	97	16.5	A	Y	35	33	−9	−9	32	28	147	132	22	23	18	14	62	63	13	10	27	23	30	35
**9**	F	8.2	7.2	102	18.5	A	Y	38	39	−5	7	29	30	145	143	29	24	−6	3	52	58	9	15	1	4	23	28
10 *	F	9.8	8.8	111	21.5	A	Y	46	42	−24	−26	22	20	152	142	25	17	−23	−17	37	45	21	25	1	9	15	25
11 *	F	8.9	7.9	96	15	S	Y	44	39	−13	−10	33	36	152	152	23	25	−13	−15	44	40	21	14	0	5	11	4
12 *	F	4.8	3.8	90	13.7	S	N	36	37	−18	−36	52	11	134	136	24	20	−14	−32	53	41	12	17	4	4	1	30
13 *	M	4.5	3.5	84	13	MS	N	26	33	−21	−33	37	18	149	156	21	21	−11	−17	60	52	5	12	10	16	23	34
14 *	M	6.8	5.8	102	16.2	S	N	37	39	−9	−10	56	42	149	141	22	23	−8	−17	50	44	15	16	1	7	6	2
15 *	M	3.8	2.8	87	16.2	S	N	34	34	−14	−19	30	42	144	143	22	21	−6	−5	56	52	12	13	8	14	26	10
16 *	F	3	2	89	12	A	N	31	30	−23	−36	24	17	155	149	10	15	−5	−2	58	52	21	15	18	34	34	35
17 *	M	4.8	3.8	88	12.6	MS	N	41	39	−18	−12	31	39	135	136	30	30	−18	−11	46	44	11	9	0	1	15	5
18 *	F	4.8	3.8	87	12.2	MS	N	28	28	−16	−20	56	42	149	147	14	12	−4	−6	56	57	14	16	12	14	0	15
19 *	M	4.5	3.5	90	13.5	MS	N	31	30	−38	−43	14	0	146	151	26	26	−23	−21	38	33	5	4	15	22	24	33

M indicates male; F, female; HSCT, hematopoietic stem cell transplantation; A, attenuated; S, Severe; MS, Most Severe; FHN, femoral head osteonecrosis; AI, acetabular index; CEA, center-edge angle; FHC, femoral head coverage; NSA, neck shaft angle (NSA); CAI, cartilaginous acetabular index; CCEA, cartilaginous center-edge angle; CFHC, cartilaginous femoral head coverage; ‘*’ indicates patients had negative value of CCEA, representing those with subluxation/dislocation of hips. The phenotype is defined by the present height; less than 25th percentile (the most severe), the 25th–75th percentile (severe), over 75th percentile (attenuated).

**Table 2:** Age-matched comparison of parameters in MPS IVA hips with the normal cohort.

Range of Age(y)	Numbers of Patients	AI (°)		CAI (°)		CEA (°)		FHC (%)		NSA (°)	
		MPS-IVA	NC †	MPS-IVA	NC†	MPS-IVA	NC ‡	MPS-IVA	NC ‡	MPS-IVA	NC §
3 to 4	3	33 ± 2	19.89	19 ± 5	8.17	−18 ± 10	23	29 ± 8	83–84	145 ± 8	145
4 to 5	6	33 ± 5	18.22	22 ± 6	7.9	−26 ± 10	23	27 ± 18	79–84	143 ± 8	145
5 to 6	1	36 ± 1	18.51	24 ± 2	8.4	−21 ± 4	23	30 ± 12	79–82	143 ± 4	142
6 to 7	2	35 ± 4	17.91	23 ± 1	8.19	−9 ± 1	24	45 ± 8	80–82	147 ± 6	142
7 to 8	1	39 ± 1	18.25	25 ± 2	8.05	−33 ± 1	26	9 ± 2	80–82	149 ± 5	142
8 to 9	4	40 ± 2	17.64	24 ± 4	8.15	−16 ± 14	25	25 ± 9	81–82	150 ± 4	142
9 to 10	2	39 ± 6	16.40	23 ± 5	7.87	−29 ± 5	25	18 ± 4	81	148 ± 4	138

NC indicates normal control; ‘†’ indicates the normal values of AI and CAI cited from Li et al. [23]; ‘‡’ indicates the normal values of CEA and FHC cited from Tsukagoshi et al. [24]; ‘§’ indicates the normal values of NSA cited from Birkenmaier et al. [25].

**Table 3:** Comparison of femoral head osteonecrosis (FHN) and non- FHN patients.

	FHN Group	No FHN Group	*p*
**Sex (no. patients)**	11	8	0.181
**Male**	8	3	
**Female**	3	5	
**Age (y)**	7.4± 2.1	4.6± 1.1	0.002 #
**Age at HSCT (y)**	6.4± 2.1	3.6± 1.1	0.002 #
**Height (cm)**	98.3± 5.0	89.6± 5.4	0.002 #
**Weight (kg)**	16.9± 2.2	13.7± 1.7	0.003 #
**AI (°)**	37.5 ± 4.0	33.4 ± 4.5	0.007 #
**CEA (°)**	−20.1 ± 11.9	−22.9 ± 10.8	0.465
**FHC (%)**	23.2 ± 10.5	31.9 ± 16.7	0.056
**NSA (°)**	146.6 ± 6.4	145.0 ± 7.0	0.478
**CAI (°)**	23.3 ± 3.5	21.1 ± 5.9	0.193
**CCEA (°)**	−9.6± 18.2	−12.5± 8.3	0.556
**CFHC (%)**	45.4 ± 12.7	49.5 ± 7.8	0.261
**∆AI**	14.2 ± 5.5	12.3 ± 4.7	0.255
**∆CEA**	10.5 ± 13.9	10.4 ± 10.0	0.965
**∆FHC**	22.2 ± 10.5	17.6 ± 14.1	0.279

‘#’ indicates a significant difference.
